# Adolescent Mental Health Priorities During the Covid-19 Pandemic

**DOI:** 10.1007/s12310-022-09547-w

**Published:** 2022-10-31

**Authors:** Tracy M. Stewart, Debi Fry, Jenny Wilson, Lesley McAra, Sarah Hamilton, Albert King, Margaret Laurie, Gillean McCluskey

**Affiliations:** 1 Moray House School of Education and Sport, University of Edinburgh, Edinburgh, EH8 8AQ Scotland, UK; 2 Edinburgh Law School, University of Edinburgh, Edinburgh, Scotland UK; 3 UNICEF-UK, London, UK; 4 Scottish Government, Edinburgh, Scotland UK

**Keywords:** Mental health, Wellbeing, Adolescents, Covid-19, School

## Abstract

Increasing evidence has shown that the Covid-19 outbreak has impacted adolescents’ mental health. Utilising a mixed-method design, the current study examined a total of 518 adolescent perspectives (60% female), in Scotland, on what *has* and *could* help their mental health in the context of Covid-19. A reflexive thematic analysis revealed three themes in relation to what *has* helped adolescents’ mental health since the Covid-19 outbreak. These related to findings about the value of: (1) engaging in recreational activities, (2) engaging with friends, and (3) the disruption to schooling. The remaining four themes related to what could have helped adolescents mental health and wellbeing since the Covid-19 outbreak. These focussed on (1) better support: in relation to mental health; school work; and communication, (2) contact with friends, and (3) more opportunities for recreational activities. Males were more likely to report recreational activities *had* helped and less likely to report better support *could* have helped. Adolescents who reached clinical threshold for depression and anxiety and those with elevated PTSD-like symptoms about Covid-19 were more likely to state more support *could* have helped, and adolescents who reached clinical threshold for depression were *less* likely to report that friends could have helped their mental health. The findings may inform mental health policy and interventions in the recovery from the Covid-19 pandemic.

In January 2020, the World Health Organisation (WHO) announced the coronavirus disease 2019 (Covid-19) outbreak as a Public Health Emergency of International Concern (PHEIC) and subsequently in March 2020 declared the outbreak a global pandemic. By mid-April 2020, with much of the world in ‘lockdown’, 86% of children and adolescents worldwide were out of school (The Lancet Child Adolescent Health, 2020). Moving forward 18 months to September 2021, the Covid-19 pandemic saw mass quarantines, school disruption, and social distancing, resulting in a number of economic, health, psychological, and social consequences (UNESCO, 2020). Even now, over 2 years on with most schools now open, UNESCO, UNICEF, and The World Bank have highlighted the detrimental consequences of the pandemic on learning and health and wellbeing (Giannini et al., 2022).

While early research focussed on transmission and treatment of the virus, and mainly in adult populations, research with children and adolescents is now emerging. For example, studies from China (Xie et al., 2020), Australia (Magson et al., 2021), and the UK (Newlove-Delgado et al., 2021) reported increases of mental ill-health in children and adolescents. Similarly, 12 longitudinal studies with pre- and during-mental health pandemic data from adolescents (mean age 15.4 years) in the USA (×10 studies), the Netherlands (×1 study), and Peru (×1 study) found depression symptoms collectively increased by 28%, although also noted that anxiety symptoms remained stable (Barendse et al., 2021). In the UK, research conducted by the Scottish Youth Parliament, YouthLink Scotland, and Young Scot (2020) reported that almost two-fifths (39%) of adolescents said they were moderately or extremely concerned about their mental wellbeing, with almost half (46%) worried about other people’s mental health. These findings are not surprising given that routines, social relationships, school learning, and leisure activities have been so severely disrupted.

A lack of connection to others can have negative consequences for physical and mental health, even leading to increased mortality (Hawkley & Cacioppo, 2010). For example, Caspi et al. (2006) reported that social isolation, feelings of loneliness and/or rejection in childhood, adolescence and young adulthood predicted cardiovascular risk factors in later young adulthood. It is also well known that adolescence is a sensitive period for social development and it has been argued that physical distancing and social deprivation may have distinctively negative consequences for adolescents (Orben et al., 2020). Adolescence marks a period of need for social connection and acceptance from peers (Andrews et al., 2020). It is a key timeframe in which adolescents spend less time with their family and more time with peers (Lam et al., 2014), where peer relationships and the development of complex social and cognitive skills are a necessity for successful transition to adulthood (Roisman et al., 2004; Rubia et al., 2006; Wood et al., 2016). Given the developmental importance of social connection, the impact of the Covid-19 pandemic may therefore be particularly harmful for adolescents. Indeed, a recent qualitative focus group study found that most adolescents reported the loss of social contact with peers during lockdown and the negative impact that had on their mental health (McCluskey et al., 2021).

Developing an understanding of adolescents’ own perspectives on their mental health since Covid-19 is vital in order to inform mental health interventions. This is in line with priorities set out in two recent multidisciplinary research position papers (Holmes et al., 2020; O’Connor et al., 2020) in which it was noted that more qualitative data were needed to inform interventions aimed at improving wellbeing and social cohesion, and where co-design with adolescents in mental health solutions was noted as a priority. O’Connor et al. (2020) highlighted the research priority to better understand the kinds of support improving long-term outcomes for children and adolescents. This is exceptionally true in the context of Covid-19 where the impact is unprecedented (O’Connor et al., 2020) and we do not yet fully understand the longer-term impact, consequences, or recovery trajectory of the pandemic (direct and indirect) for children and adolescents mental health and wellbeing.

Given its acknowledged importance, there is a surprising dearth of research which provides qualitative data examining adolescents’ experiences and perspectives on what they think has helped or could have helped their mental health in the context of Covid-19. This paper aims to bridge that key gap, by providing a detailed analysis of comments provided by adolescents in the national level study 'in isolation instead of in school' (INISS). This multi-method, multidisciplinary (Psychology, Law, Education and Sociology) and multi-industry (partnered with the Scottish Government and UNICEF-UK) mixed-method project was devised to investigate mental health and educational outcomes in adolescents in Scotland during Covid-19. The data reported in this paper pertain to one aspect of the wider INISS project. To the authors’ knowledge, this is the first comprehensive study, with a Scottish adolescent sample, to qualitatively investigate what adolescents think *has* helped or *could* have helped their mental health and wellbeing during the Covid-19 outbreak and to quantitatively investigate whether identified themes were experienced equally or differed for particular groups of adolescents. Our three main research questions were:

1. What do adolescents think *has* helped their mental health and wellbeing?
2. What do adolescents think *could* help their mental health and wellbeing?
3. Are there group differences in identified themes, depending on whether adolescents (1) received additional support in school or whether they (3) reached clinical threshold levels for depression, anxiety, or had elevated PTSD-like symptoms about Covid-19?

## Method

### Participants

A total of 899 participants, aged 14–18 years (*M*_age_ = 15.89, SD = 0.88), participated in the mixed-method INISS project, of which 518 participants (58%) provided a response to either, or both, of the two qualitative questions, pertaining to what *has* helped and what *could* have helped their mental health and wellbeing during the Covid-19 pandemic. Of the 518 participants, aged 14–18 years (*M*_age_ = 15.88, SD = 0.90), from 26 (of 32) local authority areas in Scotland, over half identified as female (60%, *n* = 311), over one-third as male (36%, *n* = 187), a small number of participants identified as non-binary (2%, *n* = 7) and one participant said they were unsure of their gender (0.2%, *n* = 1). The remaining 2% (*n* = 12) did not state their gender. Ethnicity was as follows: White (85%, *n* = 441), Asian or Asian British (6%, *n* = 32), Mixed (4%, *n* = 21), African (2%, *n* = 8), Arab (1%, *n* = 7), or Caribbean or Black (1%, *n* = 5). The remaining 1% (*n* = 5) did not specify their ethnicity. Participants were asked for information on home postcodes, allowing estimation of socio-economic status, using the Scottish Index of Multiple Deprivation (SIMD).^1^ This indicated that only 8% (*n* = 42) of participants were living in the most deprived area of the country (quintile 1), whereas 8% (*n* = 39) lived in quintile 2, 16% (*n* = 81) in quintile 3, while those living in more affluent neighbourhoods were more strongly represented [quintiles 4 (17%, *n* = 91) and quintile 5 (37%, *n* = 191)]. It was not possible to obtain SIMD scores from 14% (*n* = 74) of participants due to incomplete postcode data.

### Procedure

A research advisory group, consisting of three young people and three external research experts, advised on the INISS project. An online survey was created, which was open for responses over August and September 2020, timed to coincide with the start of the new school year. Ethical approval was obtained from Ethical approval was obtained from Moray House School of Education and Sport, University of Edinburgh. All secondary schools in Scotland were contacted via email with study details and participation access routes provided to GLOW for pupils in S4–S6 and recent school leavers (aged 14–18 years old). GLOW is the website for the Government’s national education agency, known as Education Scotland, and is accessible to all school students, teachers, and parents. Information about the project and the invitation to participate was also communicated through a variety of media, e.g. on the INISS study’s dedicated website; on Scottish Youth Parliament, YouthLink, and Pupil Inclusion Network websites.

### Measures

*What has helped and could have helped mental health and wellbeing:* Assessed by two open textbox survey questions, modelled on questions from the Academy of Medical Sciences (2020) which helped inform the framing of priorities for mental health research (Holmes et al., 2020): (1) “*Since the outbreak of Covid 19, what, if anything, has helped with your mental health and wellbeing?*” (2) “*What, if anything, could have helped with your mental health and wellbeing since the outbreak of Covid-19*?”.

*Depression and anxiety:* Assessed using the depression and anxiety subscales from the Revised Children's Anxiety and Depression Scale Short Version for Children (RCADS-25; Ebesutani et al., 2012). The RCADS-25 is a short self-report questionnaire for children and adolescents aged 8–18 years old that measures levels of depression through 10 items and broad anxiety through 15 items. Items are scored on a four-point Likert scale: 0 = Never, 1 = sometimes, 2 = Often, and 3 = Always, resulting in a range of total scores from 0 to 30. Higher total scores indicate a more severe level of depression. Clinical threshold for depression was determined by *t*-score of ≥ 70. The depression subscale had excellent reliability (*α* = 0.92) as did the anxiety subscale (*α* = 0.88).

*Avoidance and intrusion: Assessed by* an edited version of the Child Revised Impact of Events Scale short version (CRIES-8; Perrin et al., 2005) was administered. The CRIES-8 is a short self-report questionnaire for children and adolescents aged 8 years and above that measures risk for Post-Traumatic Stress Disorder (PTSD) through 8 items. Items are scored on a four-point Likert scale: 0 = Not at all, 1 = Rarely, 3 = Sometimes, and 5 = Often, resulting in a range of total scores from 0 to 40. Higher total scores indicate a more severe risk of PTSD. A total score of 17 or above was used to determine elevated levels of avoidance and intrusive thoughts. The scale had excellent reliability overall (*α* = 0.89). No changes to items were made; however, the question was pre-faced with the following text “*below is a list of comments made by people after stressful life events. [Thinking about Covid-19], please click on the responses for each item showing how frequently these comments were true for you during the past seven days. If they did not occur during that time please click the ‘not at all’ box.”* This allowed us to measure the frequency of avoidance and intrusive thoughts in relation to the Covid-19 outbreak.

*Additional support in school:* Assessed by a question devised by the project team; *“Did you have any kind of regular additional support in school before the Covid-19 outbreak?”* The item was scored as 0 = No, 1 = Not sure, 2 = Yes. A binary predictor was used for the Chi-square analyses (0 = no, 1 = yes) with ‘not sure’ responses treated as missing.

### Analytical Strategy

There were no significant differences in anxiety, depression, or PTSD-like symptoms about Covid-19, between adolescents who provided a response to the open-ended questions and those who did not, nor any significant differences in age, gender, ethnicity, or SIMD score between adolescents who provided a response to the open-ended questions and those who did not (*p* > 0.05). The qualitative data were analysed using reflexive thematic analysis (Braun & Clarke, 2006, 2019). The analysis followed the phases of reflexive thematic analysis as outlined by Braun and Clarke: familiarisation; categorisation into components through coding; identification of general themes, collation of data to themes, before producing a report. We used investigator triangulation in the analysis of the data. One author (JW) generated categories, codes, and concepts, with support from another author (TMS). Consequently, collated codes and data were abstracted into potential themes. These were then reviewed, confirmed, and refined in light of relevant literature and in iterative dialogue with the data and all authors, until all themes were agreed. This allowed for theory triangulation from multi-disciplinary professional perspectives to interpret the data, providing validity to the analysis. This formed the basis for the key themes which are discussed in detail below. Following the reflexive thematic analysis, Chi-square analyses, and where necessary, Fisher exact tests were conducted in SPSS (version 25.0) to examine potential group differences in themes. Due to low group membership in some groups (i.e. non-binary adolescents, and those unsure of their gender), only participants who identified as male and female are included in Chi-square analyses.

## Results

**Fig. 1:**
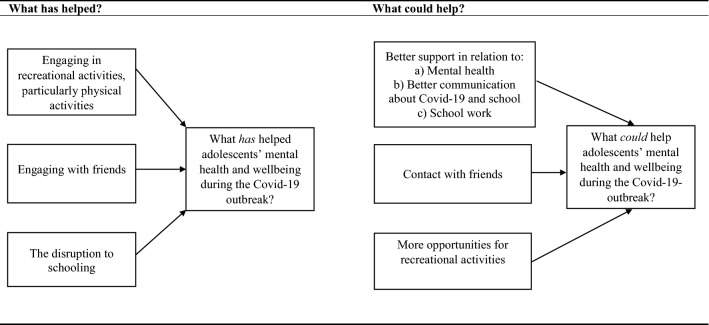
Themes: What has helped and what could help adolescent’s mental health since the Covid-19 outbreak

A total of 7 overarching themes were identified from the data (see Fig. 1). Three themes related to what *has* helped adolescents’ mental health and wellbeing since the Covid-19 outbreak. These related to findings about the value of: (1) engaging in recreational activities, (2) engaging with friends, and (3) the disruption to schooling. The remaining four themes related to what *could* have helped adolescents mental health and wellbeing since the Covid-19 outbreak: (1) better support, (2) contact with friends, and (3) opportunities for recreational activities.

### What *Did* Help Adolescents’ Mental Health Since the Covid-19 Outbreak?

A total of 504 adolescents provided a response in the open textbox when asked what helped their mental health and wellbeing since the Covid-19 outbreak. From this, 7% (*n* = 37) of participants provided responses which were unclear (e.g. *“/”; “*~~~*”; “no”; “yes”*), 2% (*n* = 11) said that they did not know what has helped their mental health since Covid-19 (e.g. *“I’m not sure if anything has”*), and 10% (*n* = 49) said that nothing has helped their mental health (e.g. *“nothing”*). Of the remaining 407, of whom 34% identified as male (*n* = 138), 63% as female (*n* = 256), 1% as non-binary (*n* = 5), and 2% (*n* = 8) preferred not to say (due to privacy concerns we do not report data for one participant who was unsure of their gender). Three broad themes were identified from the data that adolescents said *had* helped their mental health since the Covid-19 outbreak. These were (1) engaging in recreational activities, particularly physical activities, (2) engaging with friends, and (3) the disruption to schooling. Each of these is discussed below.

#### Engaging in Recreational Activities

Of the adolescents who outlined a factor that helped their mental health and wellbeing since the Covid-19 outbreak, 53% of young people (*n* = 217) referred to recreational activities. A total of 66% of males (*n* = 91), 47% of females (*n* = 120), 40% of non-binary adolescents (*n* = 2), and 50% (*n* = 4) of adolescents who preferred not to state their gender stated that recreational activities helped their mental health and wellbeing during the Covid-19 outbreak. There was a significant association between gender and engaging in recreational activities (*p* = 0.004, Fisher's exact test). Males were significantly more likely to endorse recreational activities as having helped their mental health and wellbeing during the Covid-19 outbreak, *χ*^2^ (1, *N* = 394) = 13.11, *p* < 0.001, *V* = 0.182.

Participants gave many different examples of recreational activities, from sketching, music, baking, reading, through to social media (e.g. TikTok), playing video games and walking. Interestingly, 59% of adolescents (*n* = 128) who said that a recreational activity helped their mental health, focussed on physical activities (e.g. running, yoga, walking, skateboarding, football) helpful. This is 31% of the 407 adolescents who provided a response that stated that physical activity helped their mental health and wellbeing during the Covid-19 pandemic. For example, adolescents said “*going on one walk a day actually felt like it helped and I had more time to exercise which I wouldn’t normally do at home*”—female, 16 yrs. Other adolescents referred to going to gyms (e.g. “*since gyms have reopened I have felt a bit less stressed as I have been able to exercise*”—female, 17 yrs), while others referred to the regularity of physical activity as helping their mental health and wellbeing (e.g. *“—being able to cycle more regularly”*—male, 17 yrs*;* “*doing sports and keeping active*”—male, 15 yrs; *“I began to exercise regularly everyday…”*—female, 17 yrs*).*

#### Engaging with Friends

Of the adolescents who outlined factors that helped their mental health and wellbeing since the Covid-19 outbreak, 30% highlighted that their friends had helped (*n* = 122). A total of 31% of males (*n* = 43), 30% of females (*n* = 76), 40% of non-binary adolescents (*n* = 2), and 13% (*n* = 1) of adolescents who preferred not to state their gender, stated that engaging with friends had helped their mental health and wellbeing during the Covid-19 outbreak. There was no significant association between gender and engaging with friends (*p* = 0.681, Fisher's exact test).

Of the adolescents who said their friendships helped their mental health since the Covid-19 outbreak, responses largely focussed around communication with friends (e.g. *“…talking to my friends”—*female, 15yrs; *“…contact with my friends”*—male, 15yrs; *“…calling my friends”*—female, 16yrs*;* “*chatting with friends…”*—female, 17yrs). One commented “*staying in touch with my friends, my parents do not understand how important it is to me so they try and turn wifi off, stop phone contract and confiscate my phone even though I payed for it myself”*—female, 15yrs. Almost one-third (31%) of adolescents (*n* = 38) who mentioned friends as helping their mental health specifically referred to seeing their friends or meeting their friends as helping their mental health and wellbeing during the Covid-19 pandemic. This is 9% of the 407 adolescents who provided a response that stated that seeing or meeting their friends helped their mental health and wellbeing during the Covid-19 pandemic. For example, adolescents said “*talking to my friends in real life…”*—male, 17yrs; *“…meeting with friends once restrictions eased”*—female, 17yrs; *“speaking to my friends everyday then getting to see them after such a long time….”*—female, 16yrs.

#### The Disruption to Schooling

In addition to recreational activities and friendships, some adolescents (14%, *n* = 56^2^) noted the positive impact on their mental health due to the disruption to schooling. A total of 9% of males (*n* = 12), 16% of female (*n* = 41), 20% of non-binary adolescents (*n* = 1), and 25% (*n* = 2) of adolescents who preferred not to state their gender, said that the disruption to schooling had had a positive impact on their mental health and wellbeing during the Covid-19 outbreak. There was no significant association between gender and the disruption to schooling as helping adolescents mental health and wellbeing (*p* = 0.165, Fisher's exact test).

The reasons that the disruption to schooling was noted as helping adolescents’ mental health since the Covid-19 outbreak varied. Some did not give further details, but where further detail was provided, this largely focussed around in-school difficulties and workload. For example, while some adolescents spoke about not being in school in general as having helped their mental health since the Covid-19 outbreak (e.g. *“…being away from school as this causes me to stress out”*—female, 17yrs), others noted specific social challenges (e.g. “*not being in school. A lot of my anxieties were caused by being around other people and not having anywhere to go when I became upset…*—female, 16 yrs; “*not being in a small building with many loud teenagers helped so much. The pace and workload felt far less stressful without other people around all of the time”*—non-binary, 16yrs; *“not having to go to school has boosted my self-confidence as I no longer had to worry what people thought of me…*—female, 16yrs; *“…I have been able to be myself, not my depressing school self…*—male, 15yrs). Similarly, other young people said: *“not being at school, there wasn’t really any gossip or drama, which definitely helped with my mental health”*—female, 17yrs; *“since the school closed my stress is SO much better. I don’t feel as stressed and I don’t feel like I’m being put down as much as I did when I was at school…”*—female, 17yrs; *“being away from school as it is toxic and draining”*—female, 17yrs).

Other adolescents referred to the more relaxed pace of schoolwork, reduced workload and deadlines as having helped their mental health since the Covid-19 outbreak (e.g. *“lack of school stress, no scary deadlines…”*—male, 16yrs; *“…being able to pace myself with school work*—female, 15yrs; “*being able to rest more without the stress and hard work of school”*—male, 15yrs). Others made reference to the school starting times and better sleeping schedules/getting more sleep (e.g. *“…the early start times made it hard for me to go to school at all when I am feeling down…”*—female, 16yrs), while other participants referred to the cancellation of examinations as helping their mental health (e.g. “*because exams were cancelled I felt I didn't have too much to worry about I felt relaxed for the first time since s3 [3rd year of secondary school in Scotland] onwards and it felt like a nice break from school and decreased my feeling of stress and anxiety”*—female, 17yrs; *“…before lockdown, I had a very heavy workload with my Highers [national exams taken by 16*+ *year olds in Scotland] and I had really stopped enjoying school. Having exams cancelled and no school gave me a chance to reset, figure out what I wanted to do next and make the most of things when we came back”*—female, 17yrs; *“… being able to use an average across the whole year really made me more confident in my ability to achieve what I deserved”*—female, 16yrs).

#### Group Differences in Themes

To examine group differences in themes between adolescents who did and did not (1) receive additional support in school, (2) meet clinical threshold for depression, (3) anxiety, or have (4) elevated PTSD-like symptoms about the Covid-19 outbreak, Chi-square analyses (or Fishers exact when cell count was too low) were conducted (see Table 1). All were non-significant, *p* > 0.05, suggesting no statistically significant group differences in reported themes.

**Table 1:** What has helped adolescents’ mental health since the Covid-19 outbreak, split by group membership and theme

	Recreational activities		Engaging and communicating with friends		The disruption to schooling	
	Yes: % (*n*)	No: % (*n*)	Yes: % (*n*)	No: % (*n*)	Yes: % (*n*)	No: % (*n*)
Additional support in school^1^: Yes (*n* = 54)	44 (*n* = 24)	56 (*n* = 30)	20 (*n* = 11)	80 (*n* = 43)	17 (*n* = 9)	83 (*n* = 45)
Additional support in school: No (*n* = 314)	55 (*n* = 173)	45 (*n* = 141)	33 (*n* = 104)	67(*n* = 210)	13 (*n* = 41)	87 (*n* = 273)
Depression threshold: Yes (*n* = 38)	44 (*n* = 16)	56 (*n* = 22)	26 (*n* = 10)	74 (*n* = 28)	15 (*n* = 6)	85 (*n* = 32)
Depression threshold: No (*n* = 369)	55 (*n* = 201)	45 (*n* = 168)	30 (*n* = 112)	70 (*n* = 257)	14 (*n* = 50)	86 (*n* = 319)
Anxiety threshold: Yes (*n* = 31)	42 (*n* = 13)	58 (*n* = 18)	23 (*n* = 7)	77 (*n* = 24)	13 (*n* = 4)	87 (*n* = 27)
Anxiety threshold: No (*n* = 376)	54 (*n* = 204)	46 (*n* = 172)	30 (*n* = 115)	70 (*n* = 261)	14 (*n* = 52)	86 (*n* = 324)
Elevated avoidance & intrusion: Yes (*n* = 126)	55 (*n* = 69)	45 (*n* = 57)	32 (*n* = 41)	68 (*n* = 85)	15 (*n* = 19)	85 (*n* = 107)
Elevated avoidance & intrusion: No (*n* = 281)	53 (*n* = 148)	47 (*n* = 133)	29 (*n* = 81)	71 (*n* = 200)	13 (*n* = 37)	87 (*n* = 244)

^1^37 adolescents who provided a response to what has helped their mental health and wellbeing were not sure if there received additional support in school and 2 did not provide a response to the question and have therefore been excluded from figures above

### What *Could* Have Helped Adolescents’ Mental Health Since the Covid-19 Outbreak?

A total of 395 adolescents provided a response in the open textbox as to what *could* help their mental health since the Covid-19 outbreak. From this, 40 (10%) participants provided responses that were unclear (e.g. *“/”; “.”; “yuyu”; “no” “yes”*), 45 (11%) said that they did not know what could help their mental health since Covid-19 (e.g. *“I honestly don’t know”*), and 47 (12%) said that nothing could help their mental health (e.g. *“nothin because I did not need help”*). Among the remaining 263 adolescents, of whom 32% identified as male (*n* = 85), 66% female (*n* = 172), 1% non-binary (*n* = 3), and 1% (*n* = 3) preferred not to say (due to privacy concerns we do not report data for one participant who was unsure of their gender), four broad themes were identified. These focussed on (1) better support: in relation to mental health; school work; and communication, (2) contact with friends, and (3) more opportunities for recreational activities.

#### Better Support in Relation to (a) Mental Health, (b) School Work, and (c) Better Communication About Covid-19 and School

Of the adolescents who outlined factors that *could* have helped their mental health since the Covid-19 outbreak, over one-third (43%) referred to some kind of support (*n* = 112). Where specified, responses mainly raised the need for more mental health support, support from school and better communication about Covid-19 and school. A total of 24% of males (*n* = 20), 51% of females (*n* = 87), 67% of non-binary adolescents (*n* = 2), and 100% (*n* = 3) of adolescents who preferred not to state their gender said that better support could have helped their mental health and wellbeing during the Covid-19 outbreak. There was a significant association between gender and calls for better support (*p* < 0.001, Fisher's exact test), with males significantly less likely to report that better support could have helped, *χ*^2^ (1, *N* = 257) = 17.13, *p* < 0.001, *V* = 0.258.

Over one-third (36%) of adolescents (*n* = 40) who said that a form of support could help their mental health did not directly state the type of support that could help their mental health (e.g. “*general support*”). Other young people referred to support from school, without specifying whether this referred, for example, to mental health support in school or support with school work (e.g. “*more support from school*”—female, 16yrs; “…*my school checking up on me, which they didnt [sic]*”—female, 17yrs; “*better support from the school*”—female, 17yrs). Other adolescents referred more broadly to talking to someone as a support that could have helped their mental health and wellbeing, but did not specify whether that was in relation to their mental health, school work, isolation, or something else entirely (e.g. “*someone to talk to*”).

Of those who did state the type of support that could have helped their mental health, almost one-third (31%, *n* = 35) referred specifically to mental health support^3^ (e.g. *“having someone I could talk to about my emotions sometimes…”*—female, 15yrs). This is 13% of the 263 adolescents who provided a response to this question. Adolescents outlined a variety of places and/or people that they would like support from, which ranged from GPs (e.g. “*regular phone calls from local doctors surgery* + *counselling”*—female, 16yrs) to child and adolescent mental health services (e.g. “*more helpful input from CAMHS”*—female, 16yrs). It also included references to school-based mental health professionals (e.g. *“having contact with the school counsellor*”—female, 16yrs) to more broad mental health support in school (e.g. *“… I think more support for mental health is needed in schools…, having mandatory school/wellbeing classes with other young people…”*—female, 17yrs).

Almost one quarter (24%) of adolescents (*n* = 27) who reported that more support would have helped, referred to a wish for greater clarity and further communication about Covid-19 and schooling. This was 10% of the 263 adolescents overall who provided a response to what could help their mental health and wellbeing. Adolescents noted many different types of communication, from general reassurance (e.g. *“… reassurance that things will be fine*—male, 16yrs) to better information to aid understanding of the Covid-19 outbreak (e.g. “*more understanding of the situation…”*—female, 17yrs) that could help their mental health and wellbeing. Some adolescents also said that more positively framed communication could help their mental health (e.g. “*the news and information being given out could have been displayed in a more optimistic light*”—female, 15yrs). Adolescents also noted that clearer or more frequent communication from or about schools could help their mental health (e.g. *“schools are different and I have struggled a bit with this, I feel as though some of the guidelines contradict each other and this worries me”*—female, 17yrs). Other adolescents stated that more and/or clearer information over examinations, grades, and postschool destinations could also help their mental health and wellbeing during the Covid-19 pandemic (e.g. *“…having a bit more clarity on exams and the results…—*male, 16yrs; “*more chance to provide evidence for grades…”—fe*male, 17yrs; “*more and earlier communication from school about university applications, careers advice over lockdown”*—female, 17yrs).

A smaller number of adolescents (14%, *n* = 16) who outlined a form of support as a factor that could have helped their mental health, stated that support with schoolwork could help their mental health and wellbeing (e.g. “*more support from the school, it is more difficult to learn from reading things off a screen with no explanation behind them, since starting back (most) teachers have expected us to know the things “taught” over the lockdown. This has made me stressed and my social anxiety prevents me from doing anything about it”*—female, 16yrs).

#### Contact with Friends, Including More Often, Sooner, or in Person

In addition to a need for better support, 19% of adolescents (*n* = 51) stated that contact with their friends, including more often, sooner, or in person, could have helped their mental health and wellbeing during the Covid-19 pandemic. A total of 27% of males (*n* = 23), 16% of females (*n* = 27), and 33% of non-binary adolescents (*n* = 1) said that contact with friends could help their mental health and wellbeing during the Covid-19 outbreak. There was no significant association between gender and contact with friends (*p* = 0.091, Fisher's exact test).

Adolescents highlighted that seeing their friends could have helped their mental health and wellbeing (e.g. “*I wish I could've seen my friends because they're the people I'm most close to so being away from them for many months was really upsetting and difficult for me”*—female, 17yrs), while others noted that seeing their friends sooner could help (e.g. *“being able to see friends sooner”*—male, 17yrs*; “getting to see my friends more once lockdown had started to be lifted*—*it felt like we should have been able to do more than we were allowed because there was very few cases in…”—*female, 16yrs). Others spoke about their wish to see their friends more frequently (e.g. *“I think that more regular contact with my friends, even if online or at a distance would have helped”*—male, 17yrs; *“being able to go see a friend for a little bit every couple days”*—male, 17yrs; *“lockdown being lessened quicker so I could've seen my friends more often”*—male, 15yrs).

#### Recreational Activities, Including More Often and the Re-opening of Recreational Activities

In addition to better support and friends, 16% of adolescents stated that re-opening of recreational activities, and having the opportunity to engage in them more often, could have helped their mental health and wellbeing (*n* = 43). A total of 25% of males (*n* = 21) and 13% of females (*n* = 22) said that recreational activities could help their mental health and wellbeing during the Covid-19 outbreak. There was no significant association between gender and engaging in recreational activities (*p* = 0.089, Fisher's exact test).

Adolescents highlighted that doing *more* activities could have helped their mental health and wellbeing (e.g. *“…more exercise—*male, 15yrs*”;* “*more group gaming…—*male, 17yrs*”*; *“…do more activities*”—female, 17yrs; “*more social activities*”*;* male, 16yrs) and also noted that activities remaining open or opening sooner could help their mental health and wellbeing during the Covid-19 pandemic (e.g. *“opening of sport centres sooner”*—male, 18yrs*; “the opening of a swimming pool”*—female, 15yrs*).* Over half (63%) of young people (*n* = 27) who stated that recreational activities, including the re-opening of recreational activities, could help their mental health said that being able to engage in physical activity could have helped their mental health during the Covid-19 outbreak. This is 10% of the 264 adolescents who provided a response that stated that engaging in physical activity could have helped their mental health and wellbeing during the Covid-19 pandemic. For example, adolescents said “not *being in shielding and going out for cycles”*—male, 15yrs, *“being able to play golf”*—male, 17yrs, “*more social activities”*—male, 16yrs.

#### Group Differences in Themes

To examine group differences in themes between adolescents who did and did not; (1) receive additional support in school, (2) meet clinical threshold for depression, (3) anxiety, or have (4) elevated PTSD-like symptoms about the Covid-19 outbreak, Chi-square analyses (or Fishers exact when cell count was too low) were conducted. As shown by the frequencies cross tabulated in Table 2, adolescents who reached clinical threshold for depression were significantly more likely to report that better support could help their mental health and wellbeing during the Covid-19 pandemic, *χ*^2^ (1, *N* = 263) = 15.98, *p* < 0.001, *V* = 0.246, and significantly less likely to report that seeing their friends could help their mental health and wellbeing, *χ*^2^ (1, *N* = 263) = 11.02, *p* < 0.001, *V* = 0.205. Adolescents who met clinical threshold for anxiety were significantly more likely to report that better support could help their mental health and wellbeing during the Covid-19 pandemic, *χ*^2^ (1, *N* = 263) = 13.09, *p* < 0.001, *V* = 0.223. Furthermore, adolescents who reported elevated levels of avoidance and intrusive thoughts were significantly more likely to report that better support could help their mental health since the Covid-19 outbreak, *χ*^2^ (1, *N* = 263) = 13.04, *p* < 0.001, *V* = 0.223.

**Table 2:** What could have helped adolescents’ mental health since the Covid-19 outbreak, split by group membership and theme

	Better support		Contact with friends		Recreational activities	
	Yes: % (*n*)	No: % (*n*)	Yes: % (*n*)	No: % (*n*)	Yes: % (*n*)	No: % (*n*)
Additional support in school^1^: Yes (*n* = 45)	56 (*n* = 25)	44 (*n* = 20)	16 (*n* = 7)	84 (*n* = 38)	16 (*n* = 7)	84 (*n* = 38)
Additional support in school: No (*n* = 196)	41 (*n* = 80)	59 (*n* = 116)	20 (*n* = 40)	80 (*n* = 156)	16 (*n* = 32)	84 (*n* = 164)
Depression threshold: Yes (*n* = 39)	70 (*n* = 28)**	30 (*n* = 11)**	0 (*n* = 0)**	100 (*n* = 39)	13 (*n* = 4)	87 (*n* = 35)
Depression threshold: No (*n* = 224)	38 (*n* = 84)	63 (*n* = 140)	23 (*n* = 51)	77 (*n* = 173)	17 (n = 39)	83 (*n* = 185)
Anxiety threshold: Yes (*n* = 30)	73 (*n* = 22)**	27 (*n* = 8)**	10 (*n* = 3)	90 (*n* = 27)	13 (*n* = 4)	87 (*n* = 26)
Anxiety threshold: No (*n* = 233)	238 (*n* = 90)	62 (*n* = 143)	20 (*n* = 48)	80 (*n* = 185)	17 (*n* = 39)	83 (*n* = 194)
Elevated avoidance & intrusion: Yes (*n* = 103)	56 (*n* = 58)**	44 (*n* = 45)	14 (*n* = 14)	86 (*n* = 89)	15 (*n* = 16)	85 (*n* = 87)
Elevated avoidance & intrusion: No (*n* = 160)	34 (*n* = 54)	66 (*n* = 106)	23 (*n* = 37)	77 (*n* = 123)	17 (*n* = 27)	83 (*n* = 133)

^1^20 adolescents who provided a response to what could help their mental health and wellbeing were not sure if there received additional support in school and 2 did not provide a response to the question and have therefore been excluded from figures above

**p < *.01*

## Discussion

We investigated adolescents’ views about what *has* helped and *could* have helped their mental health and wellbeing during the Covid-19 outbreak and whether identified responses varied depending on whether adolescents received additional support in school; reached clinical threshold levels for depression, anxiety, or: had elevated PTSD-like symptoms, avoidance, and intrusive thoughts about Covid-19. Our analysis identified three themes related to what *had* helped adolescents’ mental health and wellbeing since the Covid-19 outbreak. These emerged from findings about the value of: (1) engaging in recreational activities, (2) engaging with friends, and (3) the disruption to schooling. Four further themes related to what *could* have helped adolescents’ mental health and wellbeing and were drawn from findings about the need for: (1) better support, (2) contact with friends, and (3) opportunities for recreational activities.

Overall, males were more likely to report that recreational activities had helped their mental health and wellbeing during the Covid-19 outbreak and were less likely to report that better support could help their mental health. We also noted that adolescents who reached clinical threshold levels for depression and anxiety, and young people who had elevated PTSD-like symptoms about Covid-19 were more likely to state that better support could help their mental health and wellbeing during the Covid-19 pandemic. Adolescents who reached clinical threshold for depression were also less likely to report that friends could help their mental health and wellbeing.

The focus on recreational activities is supported by previous literature. It is well accepted that recreational leisure activity is generally a good indicator of health and wellbeing as it fosters self-esteem, social connectedness, both of which are linked to positive wellbeing (Kleiber & Kirshnit, 1991; Santini et al., 2020). The finding that males were more likely to report that engaging in recreational activities helped their mental health may reflect previous research that has shown sex differences in treatment and experiences of mental health difficulties (e.g. Rice et al., 2018). Further research however is warranted to investigate the reasons as to *why* males in particular found recreational activities to have helped their mental health during Covid-19.

Young people made many references to physical activity when asked what has or could have helped their mental health and wellbeing. This finding also aligns with previous research, which has demonstrated that physical activity is linked to life satisfaction in children and adolescents (e.g. García-Hermoso et al., 2020; Valois et al., 2004). Promoting space and access to recreational activities, in particular physical activities, for adolescents during any future Covid-19 outbreak may be vital for their mental health and wellbeing. This could include, for example, incorporating moderate to vigorous physical activity into classroom lessons, active lesson breaks, or active homework (e.g. Bedard et al., 2019; Martínez-López et al., 2021). This seems especially important, given that recent research has found that children and adolescents may have been spending less time engaging in physical activities, during the Covid-19 pandemic (Dunton et al., 2020).

Many adolescents highlighted that engaging with friends *has* helped, and that seeing friends more often or sooner *could* have helped their mental health and wellbeing during the Covid-19 pandemic. It is well known that adolescence is a time in development of a need for social connection and acceptance from peers (Andrews et al., 2020) and less time spent with family (Lam et al., 2014). Past research has demonstrated that lack of connection to others can have negative consequences for physical and mental health, as well as increased mortality (Caspi et al., 2006; Hawkley & Cacioppo, 2010; Orben et al., 2020). Previous research has shown that adolescents prefer informal sources of support such as speaking to friends (Raviv et al., 2000). Equipping adolescents with the necessary skills and knowledge, improving mental health literacy, and facilitating access and signposting to effectively assist a peer in distress or at risk of developing mental ill-health, will also allow young people to take action to better support mental health (Jorm, 2012). For example, in school, mental health literacy workshops could be delivered during the personal, social, and health education lessons (e.g. Patalay et al., 2017), or through peer learner lessons (e.g. Widnall et al., 2021).

It is important to note that adolescents who reached clinical threshold for depression were *less* likely to report that seeing friends more often or sooner could have helped their mental health and wellbeing. Past research has shown that adolescents with depression can be reluctant to share their feelings with friends, owing to negative experiences of having done so in the past (Anttila et al., 2015). Research has also shown that peers do not always respond in a way that is likely to facilitate appropriate support for mental health (Kelly et al., 2006; Mason et al., 2015) and it may be that adolescents who participated in the current study experiencing clinical levels of distress, would prefer more formal rather than informal sources of support.

We were interested to find that adolescents reported that the disruption to schooling *had* a positive impact on their mental health and wellbeing during the Covid-19 pandemic. This largely focussed around a break from in-school-related difficulties and high workloads. Some adolescents referred to a break from behaviours that may be indicative of experiences of bullying (e.g. “being put down”). Bullying is a worldwide problem for young people; however, recent research during the Covid-19 pandemic, albeit in Canada, has reported a reduction in bullying (Vaillancourt et al., 2021). It may be that the current study’s finding is tapping into this research. Future research however is warranted to explore this possibility. Some adolescents also referenced a more relaxed pace of schoolwork, reduced workload and deadlines as having helped their mental health since the Covid-19 outbreak. It may be that these changes led to more time for recreational leisure activities or time for themselves, as one study in Italy has already suggested (Fioretti et al., 2020).

Positive experiences that follow stressful and/or traumatic events (e.g. Covid-19 and the mitigations that followed) are not uncommon and refer to post-traumatic growth (e.g. Meyerson et al., 2011) or stress-related growth (e.g. Vaughn et al., 2009). Research with adolescents during Covid-19 has found that the use of positive reappraisal strategies (i.e. “I looked for something good in what was happening”), emotional processing (i.e. “I took time to figure out what I was feeling”), and strengths use (i.e. “during remote learning I achieved what I wanted by using my strengths”) was associated with stress-related growth (Waters et al., 2021). This suggests that supporting adolescents to validate negative experiences of the Covid-19 pandemic, as well as identifying the positive experiences, may be beneficial. Again, further research that explores these possibilities is warranted. Given that, for some adolescents, a break from school was a positive experience, it will be important for policy makers to consider this in discussions about covid recovery in the longer term and how this might inform, for example, future assessment arrangements or support structures in school.

When asked what *could* have helped adolescents’ mental health, almost half of young people referred to a need for better support. There were three distinct aspects of support which featured strongly in these responses, namely, support with their mental health, support with school work, and better communication about Covid-19 and about school itself. This echoes urgent calls from professionals to increase mental health support for children and adolescents (e.g. Idele & Banati, 2021; Loades et al., 2000). There is a large body of literature which indicates the ways in which schools can effectively structure such support, e.g. by attending to school culture, the need to create a sense of belonging, the fostering of positive student–teacher relationships, promoting young people’s voices, strengthening partnerships with related services (e.g. Child and Adolescent Mental Health Services), and strong school leadership (e.g. Barker et al., 2021; Fazel & Hoagwood, 2020; Jessiman et al., 2022; McCabe et al., 2022; Werner-Seidler et al., 2021).

Adolescents who reached clinical threshold levels for depression, anxiety, and elevated PTSD-like symptoms about Covid-19 were more likely to state that better support could have helped their mental health and wellbeing. This likely reflects current research showing that adolescents who were experiencing clinical symptoms were more severely impacted by the Covid-19 outbreak and the mitigations that followed (Zijlmans et al., 2021). Mental health support, particularly for groups of adolescents experiencing elevated clinical symptoms, should be a priority in the recovery from the Covid-19 pandemic. Specifically for adolescents experiencing clinically significant symptoms, timely access to referral pathways (both mental health services and community support), remaining sensitive to the potential long-lasting impact of the Covid-19 pandemic on adolescent mental health, and considering reasonable adjustments to educational practice may be particularly beneficial.

The finding that males were *less* likely to report that better support could help their mental health and wellbeing, aligns with past research that shows males are typically less likely to seek out mental health support (Szumilas et al., 2010). Given that males are at higher risk of suicide (e.g. Rice et al., 2018; Rodway et al., 2016), we add to calls for the development of policy, theory, and specific targeted interventions that incorporate ways to support young males (Rice et al., 2018). We also support the growing calls, now including the voices of young people, that priority should be given to increasing mental health support for children and adolescents, for it to be accessible, using face-to-face and digital methods, and from networks of parents, psychiatrists, psychologists, teachers, paediatricians, volunteers, and non-government organisations (Singh et al., 2020).

Some adolescents noted that better communication about Covid-19 and information about school (e.g. connected to examinations, grades, university applications, leaning online, returning to school after lockdown) could have helped their mental health and wellbeing. Suggestions included requests for more optimistic news and information provided in the media, to more clear and consistent messaging. Successful public health messaging typically centres around behavioural theories, with many showing that perceived susceptibility and severity of a health concern influences whether an individual will engage in action to prevent or minimise the health concern (e.g. Glanz & Bishop, 2010; Rogers, 1983; Witte, 1994). However, given the rapidly changing nature of the Covid-19 pandemic, the media, schools, as well as social conversations have been dominated by large amounts of complex and changing information. Effective communication about Covid-19, co-created and delivered with adolescents where possible, should be an essential component in the response to the pandemic, especially given that effective, but sensitive, messaging about illnesses can have benefits for psychological wellbeing (Dalton et al., 2019). Specifically for schools, clear and timely communication of expectations, changes in curriculum and examinations, with a specific emphasis on access routes to academic support is recommended. Including adolescents in the design and implementation of public health and school-based messaging may be particularly beneficial in terms of effective targeting but also because it is a fundamental human right for all, including children and adolescents, to participate in the design of policies that aim to serve them (UNICEF, 1990).

## Limitations and Conclusion

It has been suggested that pre-Covid-19 models and policies related to child and adolescent mental health may not be applicable as we move forward with Covid-19 (Singh et al., 2020). This reinforces the need to learn from the current study and its focus on adolescent perspectives. The study does however have a number of limitations. While it draws on the voices of a large number of adolescents, the use of open text boxes may have limited the richness of the data, in comparison with interview-type qualitative studies. We also were limited in the ability to follow-up responses. For example, we were unable to determine whether adolescents who responded saying “nothing” did so because they felt nothing could help, or because they felt they had good mental health and nothing more was needed. Future work, with a focus on in-depth, and reciprocal communication with adolescents in order to follow up on such points and to explore themes in greater depth would be beneficial. While our aim was to ensure that all young people had the same probability of selection and inclusion in the study, it is important to acknowledge that the final sample included an over-representation of females and a potential under-representation of young people from the most deprived data zones in Scotland. This may impact the generalisability of the findings. Furthermore, the findings, whilst important, pertain to adolescent voices from early in the pandemic and the continued inclusion of young people’s voices in relation to their own mental health should continue to be at the forefront of future research and practice. Despite these limitations, we would argue that the current findings provide a valuable starting point in learning from young people about the impact of Covid-19 on their experiences of education and mental health. We encourage scholars, practitioners, and policy makers to redouble their efforts to engage with young people, to listen to their many insightful reflections and suggestions for improvement in order to inform effective and sustained recovery from the Covid-19 pandemic.

## Footnotes

1 The Scottish Index of Multiple Deprivation (SIMD is a relative measure of deprivation derived from home postcodes, ranging from quintile 1 (the 20% most deprived data zones in Scotland) to quintile 5 (the 20% least deprived data zones in Scotland).

2 Some young people referred to getting more sleep (e.g. *“… I actually got more sleep and was a lot more happy”*) or not being in crowded places (e.g. *“not having to be in crowded places*”). It could be assumed that more sleep or not needing to be in crowded places was related to the disruption to schooling. However, we refer here to explicitly stated (as opposed to possibly implied) positive impacts of the disruption to schooling.

3 While it could be assumed that some responses refer to mental health support (e.g. *“having someone to talk to during the outbreak in lockdown”*) it was not always clear if a young person was referring to support for their mental health or their school work and thus were not included in the figures reported for young people who wanted mental health support.

## References

[CR1] Andrews JL, Foulkes L, Blakemore S-J (2020). Peer influence in adolescence: Public-health implications for COVID-19. Trends in Cognitive Sciences.

[CR2] Anttila K, Anttila M, Kurki M, Hätönen H, Marttunen M, Välimäki M (2015). Concerns and hopes among adolescents attending adolescent psychiatric outpatient clinics. Child and Adolescent Mental Health.

[CR3] Barendse, E. A. M., Flannery, J., Cavanagh, C., Aristizabal, M., Becker, S. P., Berger, EBreaux, R., Campione-Barr, N., Church, J. A., Crone, E. A., Dahl, R. E., Dennis-Tiwary, T. A., Dvorsky, M. R., Dziura, S. L., van de Groep, S., Ho, T. C., Killoren, S. E., Langberg, J. M., Larguinho, T. L., & Pfeifer, J. H. (2021). Longitudinal change in adolescent depression and anxiety symptoms from before to during the COVID-19 pandemic: A collaborative of 12 samples from 3 countries. *Journal of Research on Adolescence*. 10.31234/osf.io/hn7us 10.1111/jora.12781 PMC9349954 35799311

[CR4] Barker R, Hartwell G, Bonell C, Egan M, Lock K, Viner RM (2021). Research priorities for mental health in schools in the wake of COVID-19. Journal of Epidemiology and Community Health.

[CR5] Bedard C, St John L, Bremer E, Graham JD, Cairney J (2019). A systematic review and meta-analysis on the effects of physically active classrooms on educational and enjoyment outcomes in school age children. PLoS ONE.

[CR6] Braun V, Clarke V (2006). Using thematic analysis in psychology. Qualitative Research in Psychology.

[CR7] Braun V, Clarke V (2019). Reflecting on reflexive thematic analysis. Qualitative Research in Sport, Exercise and Health.

[CR8] Caspi A, Harrington H, Milne B, Moffit TE, Poulton R (2006). Socially isolated children 20 years later: Risk for cardiovascular disease. Archives of Pediatrics and Adolescent Medicine.

[CR9] Dalton, L., Rapa, E., Ziebland, S., Rochat, T., Kelly, B., Hanington, L., Bland, R., Yousafzai, A., Stein, A., & Communication Expert Group (2019). Communication with children and adolescents about the diagnosis of a life-threatening condition in their parent. *Lancet, 393*(10176), 1164–1176. 10.1016/S0140-6736(18)33202-1 10.1016/S0140-6736(18)33202-1 30894272

[CR10] Dunton GF, Do B, Wang SD (2020). Early effects of the COVID-19 pandemic on physical activity and sedentary behavior in children living in the U.S. BMC Public Health.

[CR11] Ebesutani C, Reise SP, Chorpita BF, Ale C, Regan J, Young J, Higa-McMillan C, Weisz JR (2012). The revised child anxiety and depression scale-short version: Scale reduction via exploratory bifactor modeling of the broad anxiety factor. Psychological Assessment.

[CR12] Fazel M, Hoagwood K (2020). School mental health: Integrating young people's voices to shift the paradigm. The Lancet: Child and Adolescent Health.

[CR13] Fioretti C, Palladino BE, Nocentini A, Menesini E (2020). Positive and negative experiences of living in COVID-19 pandemic: Analysis of Italian adolescents' narratives. Frontiers in Psychology.

[CR14] García-Hermoso H-A, Fernández-Vergara O, Oriol-Granado.  (2020). Physical activity, screen time and subjective well-being among children. International Journal of Clinical and Health Psychology.

[CR15] Giannini, S., Jenkins, R., & Saaverdra, J. (2022). Where are we on education recovery? United Nations Children’s Fund (UNICEF) New York. https://www.unesco.org/en/covid-19/education-response

[CR16] Glanz K, Bishop DB (2010). The role of behavioral science theory in the development and implementation of public health interventions. Annual Review of Public Health.

[CR17] Hawkley LC, Cacioppo JT (2010). Loneliness matters: A theoretical and empirical review of consequences and mechanisms. Annals of Behavioral Medicine.

[CR18] Holmes EA, O’Connor RC, Perry VH, Tracey I, Wessely S, Arseneault L, Ballard C, Christensen H, Cohen Silver R, Everall I, Ford T, John A, Kabir T, King K, Madan I, Michie S, Przybylski AK, Shafran R, Sweeney A, Bullmore E (2020). Multidisciplinary research priorities for the COVID-19 pandemic: A call for action for mental health science. Lancet Psychiatry.

[CR19] Idele P, Banati P (2021). We are all in this together: COVID-19 and a call to action for mental health of children and adolescents. Frontiers in Psychiatry.

[CR20] Jessiman P, Kidger J, Spencer L, Geijer-Simpson E, Kaluzeviciute G, Burn A-M, Leonard N, Limmer M (2022). School culture and student mental health: A qualitative study in UK secondary schools. BMC Public Health.

[CR21] Jorm AF (2012). Mental Health Literacy: Empowering the community to take action for better mental health. American Psychologist.

[CR22] Kelly CM, Jorm AF, Rodgers B (2006). Adolescents' responses to peers with depression or conduct disorder. Australian and New Zealand Journal of Psychiatry.

[CR23] Kleiber DA, Kirshnit CE, Diamant L (1991). Sport involvement and identity formation. Mind-body maturity: Psychological approaches to sports, exercise, and fitness.

[CR24] Lam CB, McHale SM, Crouter AC (2014). Time with peers from middle childhood to late adolescence: Developmental course and adjustment correlates. Child Development.

[CR25] Loades ME, Chatburn E, Higson-Sweeney N, Reynolds S, Shafran R, Brigden A, Linney C, McManus N, Borwick C, Crawley E (2020). Rapid systematic review: The impact of social isolation and loneliness on the mental health of children and adolescents in the context of COVID-19. Journal of the American Academy of Child and Adolescent Psychiatry.

[CR26] Magson NR, Freeman JY, Rapee RM, Richardson CE, Oar EL, Fardouly J (2021). Risk and protective factors for prospective changes in adolescent mental health during the COVID-19 pandemic. Journal of Youth and Adolescence.

[CR27] Martínez-López EJ, Ruiz-Ariza A, de la Torre-Cruz M, Suárez-Manzano S (2021). Alternatives of physical activity within school times and effects on cognition. A systematic review and educational practical guide. Psicología Educativa.

[CR28] Mason RJ, Hart LM, Rossetto A, Jorm AF (2015). Quality and predictors of 696 adolescents׳ first aid intentions and actions towards a peer with a mental health 697 problem. Psychiatry Research.

[CR29] McCabe EM, Davis C, Mandy L, Wong C (2022). The role of school connectedness in supporting the health and wellbeing of youth: Recommendations for school nurses. NASN School Nurse.

[CR30] McCluskey G, Fry D, Hamilton S, King A, Laurie M, McAra L, Stewart TM (2021). School closures, exam cancellations and isolation: The impact of Covid-19 on young people’s mental health. Emotional and Behavioural Difficulties.

[CR31] Meyerson DA, Grant KE, Carter JS, Kilmer RP (2011). Posttraumatic growth among children and adolescents: A systematic review. Clinical Psychology Review.

[CR32] Newlove-Delgado, T., McManus, S., Sadler, K., Thandi, S., Vizard, T., Cartwright, C., Ford, T., & Mental Health of Children and Young People Group. (2021). Child mental health in England before and during the COVID-19 lockdown. *Lancet Psychiatry, 8*(5), 353-354. 10.1016/S2215-0366(20)30570-8 10.1016/S2215-0366(20)30570-8 PMC8824303 33444548

[CR33] O’Connor DB, Aggleton JP, Chakarabarti B, Cooper CL, Creswell C, Dunsmuir S, Fiske ST, Gathercolde S, Gough B, Ireland JL, Jones MV, Jowett A, Kagan C, Karanika-Murray M, Kaye LK, Kumari V, Lewandowsky S, Lightman S, Malpass D, Armitage CJ (2020). Research priorities for the COVID-19 pandemic and beyond: A call to action for psychological science. British Journal of Psychology.

[CR34] Orben A, Tomova L, Blakemore S-J (2020). The effects of social deprivation on adolescent development and mental health. Lancet Child & Adolescent Health.

[CR35] Patalay P, Annis J, Sharpe H, Newman R, Main D, Ragunathan T, Parkes M, Clarke K (2017). A pre-post evaluation of OpenMinds: A sustainable, peer-led mental health literacy programme in Universities and secondary schools. Prevention Science.

[CR36] Perrin S, Meiser-Stedman R, Smith P (2005). The children's revised impact of event scale (CRIES): Validity as a screening instrument for PTSD. Behavioural and Cognitive Psychotherapy.

[CR37] Raviv A, Sills R, Raviv A, Wilansky P (2000). Adolescents’ help-seeking behaviour: The difference between self- and other-referral. Journal of Adolescence.

[CR38] Rice SM, Purcell R, McGorry PD (2018). Adolescent and young adult male mental health: Transforming system failures into proactive models of engagement. Journal of Adolescent Health.

[CR39] Rodway C, Tham S-G, Ibrahim S, Turnbull P, Windfuhr K, Shaw J, Kapur N, Appleby L (2016). Suicide in children and young people in England: A consecutive case series. Lancet Psychiatry.

[CR40] Rogers RW, Cacioppo J, Petty RE (1983). Cognitive and physiological processes in fear appeals and attitude change: A revised theory of protection motivation. Social psychophysiology.

[CR41] Roisman GI, Masten AS, Coatsworth JD, Tellegen A (2004). Salient and emerging developmental tasks in the transition to adulthood. Child Development.

[CR42] Rubia K, Smith AB, Woolley J, Nosarti C, Heyman I, Taylor E, Brammer M (2006). Progressive increase of frontostriatal brain activation from childhood to adulthood during event-related tasks of cognitive control. Human Brain Mapping.

[CR43] Santini ZI, Stougaard S, Koyanagi A, Ersbøll AK, Nielsen L, Hinrichsen C, Madsen KR, Meilstrup C, Stewart-Brown S, Koushede V (2020). Predictors of high and low mental well-being and common mental disorders: Findings from a Danish population-based study. European Journal of Public Health.

[CR44] Singh S, Roy D, Sinha K, Parveen S, Sharma G, Joshi G (2020). Impact of COVID-19 and lockdown on mental health of children and adolescents: A narrative review with recommendations. Psychiatry Research.

[CR45] Szumilas M, Kutcher S, LeBlanc JC, Langille DB (2010). Use of school-based health centres for mental health support in Cape Breton, Nova Scotia. The Canadian Journal of Psychiatry.

[CR100] The Academy of Medical Sciences. (2020). *Survey results: Understanding people’s concerns about the mental health impacts of the COVID-19 pandemic*. http://www.acmedsci.ac.uk/COVIDmentalhealthsurveys

[CR46] The Lancet Child Adolescent Health (2020). Prioritising children's rights in the COVID-19 response. The Lancet. Child & Adolescent Health.

[CR47] The Scottish Youth Parliament, YouthLink Scotland and Young Scot. (2020). *LockdownLowdown*—*What young people in Scotland are thinking about COVID-19.* https://syp.org.uk/our-work/political-work/covid-19-lockdown-lowdown/

[CR48] UNESCO. (2020). *What Have We Learnt? Overview of Findings from a Survey of Ministries of Education on National Responses to COVID-19*. UNES. https://unesdoc.unesco.org/ark:/48223/pf0000374702

[CR49] UNICEF. (1990). *Convention on the rights of the child (1989)*. https://downloads.unicef.org.uk/wp-content/uploads/2010/05/UNCRC_PRESS200910web.pdf?_ga=2.78590034.795419542.1582474737-1972578648.1582474737

[CR50] Vaillancourt T, Brittian H, Krysgman A, Farrell AH, Landon S, Pepler D (2021). School bullying before and during COVID-19: Results from a population based randomized design. Aggressive Behavior.

[CR51] Valois RF, Zullig KJ, Huebner ES, Drane JW (2004). Physical activity behaviors and perceived life satisfaction among public high school adolescents. Journal of School Health.

[CR52] Vaughn AA, Roesch SC, Aldridge AA (2009). Stress-related growth in racial/ethnic minority adolescents: Measurement structure and validity. Educational and Psychological Measurement.

[CR53] Waters L, Allen K, Arslan G (2021). Stress-related growth in adolescents returning to school after COVID-19 school closure. Frontiers in Psychology.

[CR54] Werner-Seidler A, Spanos S, Calear AL, Perry Y, Torok M, O’Dea B, Christensen H, Newby JM (2021). School-based depression and anxiety prevention programs: An updated systematic review and meta-analysis. Clinical Psychology Review.

[CR55] Widnall E, Dodd S, Simmonds R, Bohan H, Russell A, Limmer M, Kidger J (2021). A process evaluation of a peer education project to improve mental health literacy in secondary school students: Study protocol. BMC Public Health.

[CR56] Witte K (1994). Fear control and danger control: A test of the extended parallel process model (EPPM). Communication Monographs.

[CR57] Wood D, Crapnell T, Lau L, Bennett A, Lotstein D, Ferris M, Kuo A (2016). Emerging adulthood as a critical stage in the life course. Handbook of Life Course Health Development.

[CR58] Xie X, Xue Q, Zhou Y, Zhu K, Liu Q, Zhang J, Song R (2020). Mental health status among children in home confinement during the coronavirus disease 2019 outbreak in Hubei province, China. JAMA Pediatrics.

[CR59] Zijlmans J, Teela L, van Ewijk H, Klip H, van der Mheen M, Ruisch H, Luijten M, van Muilekom M, Oostrom K, Buitelaar J, Hoekstra PJ, Lindauer R, Popma A, Staal W, Vermeiren R, van Oers HA, Haverman L, Polderman TJC (2021). Mental and social health of children and adolescents with pre-existing mental or somatic problems during the COVID-19 pandemic lockdown. Frontiers in Psychiatry.

